# Different novelties revealed by infants’ pupillary responses

**DOI:** 10.1038/s41598-018-27736-z

**Published:** 2018-06-22

**Authors:** Yi-Chuan Chen, Gert Westermann

**Affiliations:** 1 Department of Psychology, Lancaster University, Lancaster, LA1 4YF UK; 2 Department of Medicine, Mackay Medical College, No.46, Sec.3, Zhongzheng Rd., Sanzhi Dist., New Taipei City, 252 Taiwan

## Abstract

To account for infants’ perceptual and cognitive development, the constructivist model proposes that learning a new object depends on the capability of processing simpler lower-level units, and then integrating these units into more complex higher-level units based on their relationships, such as regular co-occurrence. Here, we demonstrate that the process of associating visual and auditory attributes to build a new multisensory object representation is not only observed in the course of development, but also in the course of infants’ in-the-moment information processing. After a brief familiarization session of learning two pairs of novel audiovisual stimuli, 15-month-old infants showed two components in pupil dilations over time: A rapid dilation was observed when processing perceptually novel compared to familiar stimuli, and a slower dilation was observed when processing novel combinations of familiar stimuli. However, in 10-month-old infants, only the effect elicited by novel stimuli was observed. Our results therefore demonstrate that detecting perceptual novelty occurred earlier than detecting association novelty in infants’ information processing. These results support the view that infants perceive newly-learned objects by processing their constituent attributes and then integrating these components, as suggested by the constructivist model.

## Introduction

How developing infants process incoming information and acquire new knowledge in order to understand the world is a fascinating question. One dominant view, the constructivist model, proposes a hierarchical system: Infants develop capabilities of processing simpler and lower-level units, and then learn to combine these units in terms of their relationships to construct progressively more complex and higher-level units^1–4^. Typically, infants tend to utilize the highest-level unit that is available for information processing. Nevertheless, when the infants’ cognitive system is overloaded, the highest-level units are broken down and lower-level units are used instead^1,2^. These information-processing principles are suggested to be domain-general and they have been used to understand infants’ development in various aspects of cognitive processing, such as association learning^5–8^, category formation^9,10^, and causal relations^11,12^. The notion of the constructivist model is consistent with current influential theories of visual object recognition based on the integration of featural components in hierarchical feedforward processing in human adults^13–15^.

A classic experimental procedure to investigate infants’ development of association learning utilizes looking time measures embedded in a switch paradigm. These measures are based on the finding that infants typically prefer to look at novel or surprising stimuli rather than familiar stimuli (novelty preference)^16^. Specifically, in a switch paradigm, infants are familiarized with two objects composed of specific features within one sensory modality (such as visual shapes and colours) or across sensory modalities (such as visual objects and spoken words). At the subsequent test the infants are then presented with three types of stimuli: A *familiar* object that was seen during familiarization (familiar trial), a *novel* object with novel features (novel trial), and, crucially, an object comprising previously seen features but in novel combinations (*switched* trial, such as the visual shape of object A with the color of object B). Longer looking time in the novel trial than in the familiar trial allows researchers to ascertain that infants indeed show the novelty preference. Importantly, if infants have learned the associations between the features during the familiarization phase, their looking time in the switched trial should also be longer than in the familiar trial. Longer looking in the novel than in the familiar trial, and longer looking in the switched than in the familiar trial, have been reported in infant studies addressing the detection of visual feature correlations in 7- and 10-month-olds^5,6^, associating object motion and vowel sounds in 7-month-olds^17^, linking objects with actions directed at them in 10-months olds^8^, and word-object mappings in 14-month-olds^7^. On the other hand, younger infants may only demonstrate longer looking in the novel trial rather than in the switched trial as compared to the familiar trial, suggesting that they are only sensitive to the changing of familiar features rather than their combinations. This developmental time course of association learning is consistent with the simple-to-complex process proposed by the constructivist model.

Research utilizing looking time measures in the switch paradigm has provided an extensive understanding of infants’ development of association learning^18–20^. However, due to the fact that the looking time in a trial is a macro-analysis of infants’ behaviour^18^, it is not possible to further separate the infants’ responses to different types of novelty. Specifically, longer looking in the *novel* trial than in the *familiar* trial is mainly attributable to infants perceiving new stimuli (called *perceptual novelty* hereafter), whereas longer looking in the *switched* trial than in the *familiar* trial is attributable to infants detecting the incongruence of just-learned pairings (called *association novelty* hereafter). The mechanisms underlying the detection of perceptual and association novelty in the novel and switched trial, respectively, are essentially different. Nevertheless, whether and how looking times would be different in the novel and switched trials is rarely discussed. Here we used pupil dilation as a more fine-grained measure in order to provide a better understanding of infants’ in-the-moment information processing during association learning.

Pupil dilation has been demonstrated to be a sensitive and implicit measure of human cognitive functions^21–23^, and it has begun to be used in infant studies^24–27^. Human pupils dilate not only when the ambient light is dim, but also when encountering something novel because processing new as compared to familiar stimuli is more arousing and cognitively demanding^21,22^. More critically, pupil dilation provides a time-sensitive, continuous measure of in-the-moment information processing. Pupillary responses therefore provide a possible measure to separate the time-course of perceptual vs. association novelty in the switch paradigm.

**Figure 1:**
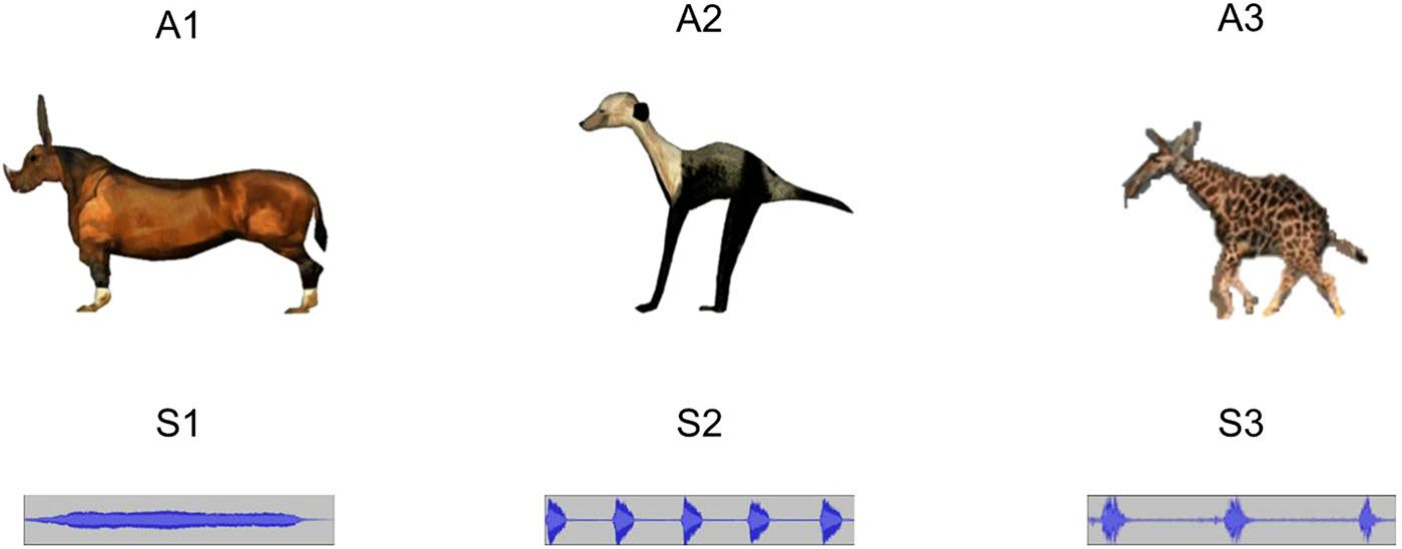
Stimuli. Three novel animals and unfamiliar sounds used in the present study. The animals were created and animated using the Animation:Master software (Hash, Inc).

In the present study, we aimed to demonstrate that the simple-to-complex hierarchical processing principles proposed in the constructivist model not only occur over the developmental time course but also in infants’ in-the-moment information processing. To do so, we measured pupil dilation (as well as looking time) in the switch paradigm: In a familiarization phase, infants repeatedly watched two novel animals, each producing a characteristic sound (e.g., A1-S1 and A2-S2, see Fig. 1). In the test phase, three types of trials were presented: The old stimuli in the same pairings (*familiar* trials), the old stimuli in a new pairing by swapping the sounds (*switched* trials, e.g., A1-S2 and A2-S1), or completely novel stimuli (*novel* trial, e.g., A3-S3).

The constructivist model suggests that the ability to integrate multisensory features of an object and the capability to maintain higher-level representations develop in infancy. Hence, the prediction was that both younger and older infants’ pupils should dilate more in the novel than in the familiar trials (and perhaps than in the switched trials as well), suggesting an effect contrasting the processing of novel vs. familiar stimuli. More critically, only the older infants’ pupils should dilate more in the switched than in the familiar trials, suggesting an effect contrasting the processing of new vs. familiar pairings of already-known stimuli^1,28^. We also expected that pupil dilation would provide a more detailed and sensitive measure regarding infants’ cognitive development than looking times which simply provide a macro measure in a trial^22,24^.

More importantly, following the hierarchical processing principles proposed by the constructivist model, pupil dilation in response to the presentation of novel stimuli (perceptual novelty) should occur earlier than that in response to the new pairing of old stimuli (association novelty) in the time-course of information processing. Hence, we predicted two pupillary response components occurring at different times following stimulus onset: a rapid response to perceptual novelty, and a slower response to association novelty.

## Results

### Proportion of looking time

In the familiarization phase, the proportion of looking time was reduced in both 10-month-olds (N = 14) and 15-month-olds (N = 11) when comparing the 1^st^ and 4^th^ block (Table 1). The rate of reduction was similar in the two age groups (*t*(22) = 0.23, *p* = 0.82, Hedges’ *g*_*s*_ = 0.09^29^). Hence, infants aged 10 and 15 months demonstrated a similar looking time performance in the familiarization phase.

**Table 1:** Proportion of looking time (%) during the familiarization phase.

Age	10-month-olds				15-month-olds			
Block	1	2	3	4	1	2	3	4
Mean	91.9	87.3	86.5	80.0	91.3	89.9	84.7	78.2
*SE*	2.2	4.7	3.7	4.7	3.2	3.2	4.9	5.7
Block 1 vs. 4	*t*(13) = 2.54, *p* < 0.05 Hedges’ *g*_*av*_ = 0.81				*t*(10) = 2.58, *p* < 0.05Hedges’ *g*_*av*_ = 0.78			

In the test phase (see Table 2), nevertheless, the proportion of looking time was significantly different between the familiar, switched, and novel conditions only in the 15-month-olds (*F*(2,20) = 5.33, *p* < 0.05, ${\eta }_{p}^{2}$ = 0.35). Planned *post*-*hoc t*-tests demonstrated that, in the 15-month-olds, the proportion of looking time was higher in the novel than in the familiar condition (*t*(10) = 3.43, *p* < 0.005, Hedges’ *g*_*av*_ = 1.37), suggesting that these infants detected the perceptual novelty of new stimuli. The proportion of looking time was higher in the switched than in the familiar condition, but the difference failed to reach statistical significance (*t*(10) = 1.29, *p* = 0.11, Hedges’ *g*_*av*_ = 0.50). In the 10-month-olds, the proportion of looking time failed to reach statistical significance between the familiar, switched, and novel conditions (*F*(2,26) = 1.26, *p* = 0.30, ${\eta }_{p}^{2}$ = 0.09). Nevertheless, the planned comparisons demonstrated that the proportion of looking time was higher in the novel than in the familiar condition (*t*(13) = 1.77, *p* < 0.05, Hedges’ *g*_*av*_ = 0.50), whereas it remained non-significant between the familiar and switched conditions (*t*(13) = 0.56, *p* = 0.29, Hedges’ *g*_*av*_ = 0.19). Note that the *p* values for the tests of looking time measures reported here are results of one-tailed *t*-test since there were clear assumptions that the proportion of looking time should be higher in the novel than in the familiar condition, and higher in the switched than in the familiar condition. The *p* values were not corrected since the comparison between the novel and familiar conditions was the only *t*-test reaching significance in both 10- and 15-month-olds, so the family-wise Type-I error rate (α) remained well controlled at the level of 0.05.

**Table 2:** Proportion of looking time (%) during the test phase.

Age	10-month-olds			15-month-olds		
Condition	Familiar	Switched	Novel	Familiar	Switched	Novel
Mean	89.5	92.0	95.2	83.9	89.5	96.1
*SE*	3.7	2.8	1.5	3.3	3.0	1.2

### Pupil dilation

The pupil diameter data were time locked to the onset of the sound (3680 ms after the onset of the test trial). Hence, the pupillary responses should be mainly elicited by the presentation of the new sound (by comparing the novel vs. familiar/switched condition) and/or the new relationship between the visual and auditory stimulus (by comparing the switched vs. familiar condition).

**Figure 2:**
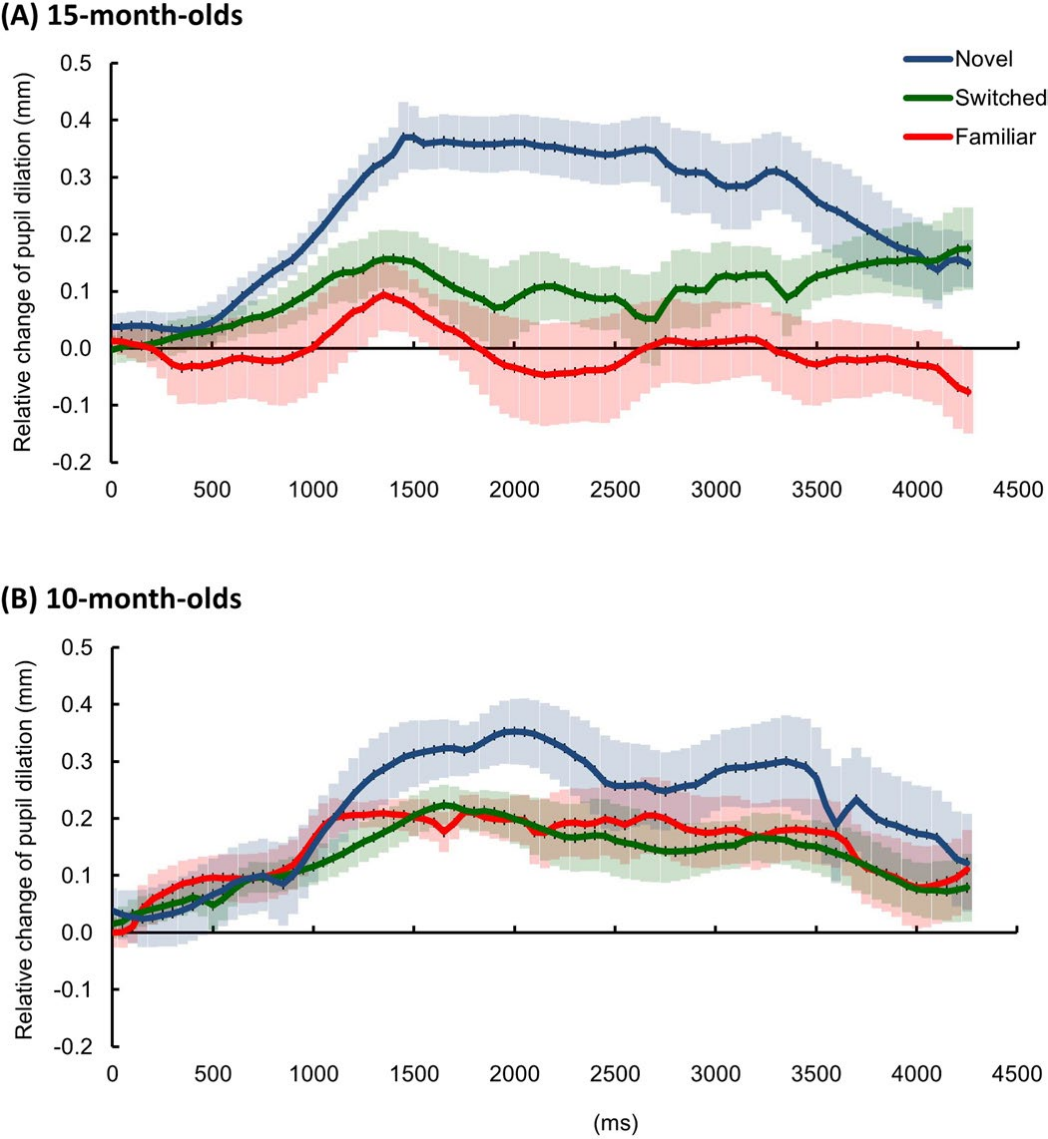
Pupil dilation results. Mean relative change of the pupil size in each condition for (**A**) 15-month-olds and (**B**) 10-month-olds, respectively. The pupil size data were locked to the onset of the sound, and the mean pupil size over the 1000 ms window before sound onset were the baseline used to normalize the data in each condition for each participant. The data from sound onset to the end of the video (4300 ms in total) were divided into 86 50-ms time bins. The shaded areas represent ±1 SE of the mean in each time bin in each condition.

**Figure 3:**
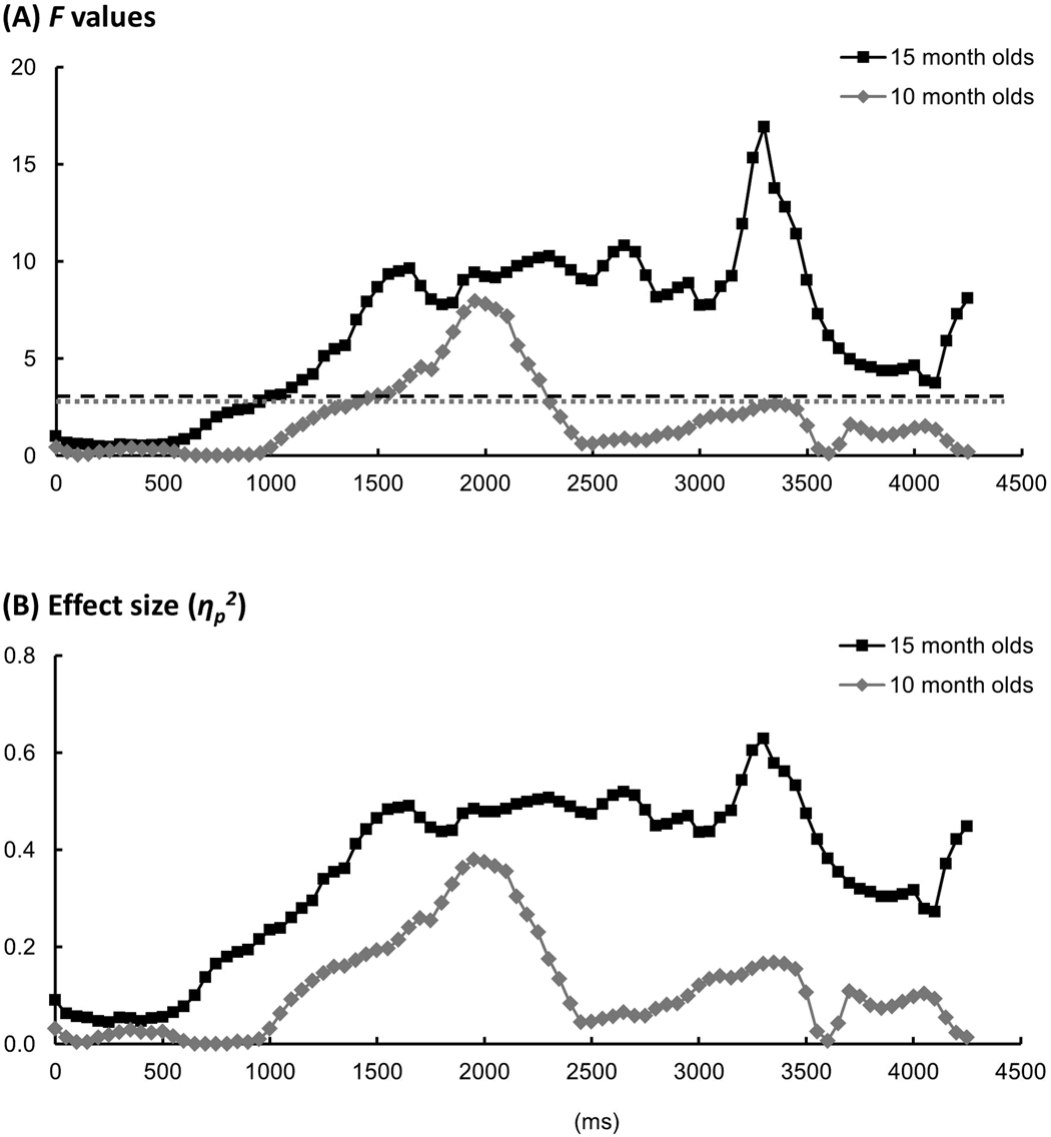
Results of the one-way ANOVA. (**A**) The *F* values in each time bin. The black dashed line represents the critical value of the *F* test for 15-month-olds (*F*(2,20) = 3.50), and the grey dotted line represents the critical value of the *F* test for 10-month-olds (*F*(2,26) = 3.37). (**B**) The effect size (${\eta }_{p}^{2}$) in each time bin.

Between the three test conditions, infants aged 15 months demonstrated significantly different pupil dilations starting from 1150 ms after sound onset to the end of the trial (Figs 2A and 3, all *F*(2,20) ≥ 3.75, *p*s < 0.05, ${\eta }_{p}^{2}$ ≥ 0.28; Monte Carlo *p*s ≤ 0.044). *Post*-*hoc t*-tests (Bonferroni corrected) revealed greater pupil dilation in the novel than in the familiar condition from 1300 to 2800 ms after sound onset (all *t*(10) ≥ 3.24, *p*s < 0.05, Hedges’ *g*_*av*_ ≥ 1.17^29^; Monte Carlo *p*s ≤ 0.022), and greater dilation in the novel than in the switched condition from 1850 to 2850 ms after sound onset (all *t*(10) ≥ 3.16, *p*s < 0.05, Hedges’ *g*_*av*_ ≥ 1.23; Monte Carlo *p*s ≤ 0.002). These results suggest an effect of *perceptual novelty* (i.e., greater pupil dilation to novel than to familiar stimuli). Moreover, greater pupil dilation was observed in the *switched* than in the *familiar* condition from 3950 to 4300 ms after sound onset (all *t*(10) ≥ 2.92, *p*s < 0.05, Hedges’ *g*_*av*_ ≥ 0.81; Monte Carlo *p*s ≤ 0.046). This result suggests an effect of *association novelty* (i.e., greater pupil dilation to new than to familiar pairings of known components). When comparing these results, the effect of association novelty started 2650 ms later than that elicited by perceptual novelty.

Infants aged 10 months demonstrated differing pupil sizes in the three conditions from 1700 to 2200 ms after sound onset (Figs 2B and 3, all *F*(2,26) ≥ 4.44, *p*s < 0.05, ${\eta }_{p}^{2}$ ≥ 0.25; Monte Carlo *p*s ≤ 0.021). *Post*-*hoc t*-tests demonstrated that this effect arose from greater pupil dilation in the novel than in the familiar condition (2000–2100 ms after sound onset, all *t*(13) ≥ 2.94, *p*s < 0.05, Hedges’ *g*_*av*_ ≥ 0.77; Monte Carlo *p*s ≤ 0.003), and greater dilation in the novel than in the switched condition (1950–2100 ms after sound onset, all *t*(13) ≥ 2.92, *p*s < 0.05, Hedges’ *g*_*av*_ ≥ 0.72; Monte Carlo *p*s ≤ 0.010). In contrast to the 15-month-old infants, there was no difference between the familiar and switched conditions (all *t*(13) ≤ 1.64, *p*s > 0.12, Hedges’ *g*_*av*_ ≤ 0.56).

Together, in the commonly-used index of the proportion of looking time, only the perceptual novelty effect was observed in both 10- and 15-month-olds. In contrast, by measuring pupillary responses, we demonstrated that infants at 15 months of age processed perceptual novelty as well as association novelty; and critically, these two effects were dissociated in terms of their time courses. In contrast, the 10-month-old infants only demonstrated responses to perceptual novelty, and this occurred later than the same effect observed in the 15-month-olds.

## Discussion

Here we use the novel index of pupillary responses to demonstrate a more fine-grained effect of infants’ association learning: While 10-month-old infants were able to detect the presentation of new stimuli (perceptual novelty), only at the age of 15 months did infants detect novel pairings of familiar features presented crossmodally (i.e., association novelty)^7,8,28^. Specifically, 10-month-old infants were only able to represent individual animals and their calls rather than their relationships, perhaps due to their limited cognitive resources^1,2^. In contrast, 15-month-old infants were able to represent both unimodal stimuli as well as their associations. We further found that processing of perceptual novelty occurred later in the 10-month-old (around 2000 ms after stimulus onset) than in the 15-month-old infants (around 1300 ms after stimulus onset).

More importantly, we demonstrate that, in 15-month-old infants, the time courses of responses to perceptual and association novelty were dissociated. The rapid pupillary response to novel stimuli and the slower pupillary response to the novel combinations of familiar stimuli in this age group suggest that the constituent unimodal stimuli were processed first and then combined into a multisensory representation. Such a binding process relies on the regular co-occurrence of the two unimodal stimuli and is underpinned by the mechanism of association learning. Once an associative connection is established, it is plausible that the congruency between the visual and auditory stimuli can be detected when they interact during feedforward information processing^30^. Our results are therefore consistent with the constructivist model but extend this notion from describing developmental differences across ages to also accounting for infants’ in-the-moment information processing.

The present study provides a critical advance in methodology for infant studies. In the looking time measure we only observed the effect of perceptual novelty between familiar and novel stimuli in both age groups. In contrast, we observed three differentiable effects in the pupil dilation measures: the rapid effect of perceptual novelty between familiar and novel stimuli in both 10- and 15-month-olds, as well as the slow effect of association novelty between familiar and switched pairings in 15-month-olds. Such dissociations between pupil dilation and looking time measures have repeatedly been reported in previous research^24–27^. Our results agree with this increasing evidence suggesting that, for the purpose of understanding infants’ cognitive processing, pupil dilation is a more sensitive measure than proportion of looking time in terms of temporal characteristics, mechanisms, and optimal experimental designs (see below).

Pupil dilation measures provide a continuous analysis of information processing over time, so that an event-related effect can be revealed at a particular time. In contrast, looking time measures are typically cumulative, and a transient effect may be diluted over time^22,23^. These facts can explain the results that the association novelty effect in 15-month-olds was only observed in the pupil dilation and not in looking times. That is, in the familiar and switched trials, the similar looking responses to the old stimuli likely masked the later different looking responses to the familiar vs. new pairings of the stimuli when both responses were merged into a single behavioural index. Similarly, when comparing the performance in the novel and the switched trials, which is rarely discussed in the literature, the different looking responses to the new vs. old stimuli were perhaps diluted by the subsequently similar responses to novel stimulus pairings. The first, and the most critical, advantage in using pupil dilation measures therefore lies in that the time course of transient effects elicited by a particular event can be clearly revealed.

The cognitive mechanism underpinning the pupil dilation measure is that human pupil size is positively correlated with increasing mental activity (such as in a high arousal state^21^, conducting a high cognitive-load task^22^ or retrieval from episodic memory^31^) irrespective of the conscious level of the information processing^21^. Looking time measures, on the other hand, are mainly based on the assumption of novelty preference; nevertheless, some infants may show *familiarity preference* in the same experimental design if they were not habituated to the stimuli, leading to a cancelled-out or uninterpretable result between participants^19^. Hence, pupil dilation seems to be a more reliable measure since its correlation to mental processing load is always positive.

Finally, in order to collect high-quality data for pupillary responses (i.e., encouraging the participants to continuously watch the stimuli), the experimental designs in pupil dilation studies are typically short and highly attractive. On the other hand, in order to ensure that the novelty preference for looking time measures can be observed, reaching a habituation criterion in the preceding learning/familiarization session is necessary, a procedure that typically takes several minutes^32^. The experimental designs for pupil dilation measures therefore reduce the influence of the participants’ fatigue, inattention, and impatience^22^. Hence, when using pupil dilation measures, as demonstrated in the present study, a long habituation/adaptation procedure is not necessary to demonstrate pupil dilation responses to various kinds of novelties.

In sum, we demonstrated that pupil dilation is a powerful measure to understand the time-course of infants’ learning and processing of multisensory objects. Specifically, the process of combining simple unimodal units to construct more complex multimodal units as suggested by the constructivist model can be demonstrated in the course of the development^33^, but critically, as we show here, also in the moment-to-moment time-course of infants’ information processing.

## Methods

### Participants

Two age groups of infants were tested: 10 months old (N = 18, 11 males, mean age = 10.1 months, range = 9.6 to 10.6 months), and 15 months old (N = 16, 7 males, mean age = 15.1 months, range = 14.8 to 15.6 months). An additional three 10-month-olds and five 15-months-olds were tested but excluded because they did not complete the experiment due to fussiness or unwillingness to watch (one 10-month-old and four 15-months-olds) or due to low quality of eye tracking because of their bright iris or failure to calibrate (two 10-month-olds and one 15-months-old). For all participants, their parents were provided with a brief explanation of the study and provided written informed consent. The age of the infants was chosen based on the results of previous studies showing that the ability to rapidly learn arbitrary pairings of audiovisual stimuli (used in the current study) develops at around 12 months old^28^, which is later than the age to rapidly learn non-arbitrary pairings^34,35^. All of the procedures were carried out in accordance with the Declaration of Helsinki. The protocol of this study was approved by the ethics committee at Lancaster University.

### Stimuli and Apparatus

The visual stimuli were presented on a 22-inch LCD monitor controlled by a personal computer. An eye tracker (Tobii X120, Tobii Technology) positioned below the monitor was used to record the infants’ diameter of pupils and eye movements. The infants sat on their caregiver’s lap, approximately 65 cm from the monitor in a dimly-lit chamber.

In order to maintain a high recording rate of pupillary responses (i.e., a high looking time), we designed a short experimental session and created highly attractive stimuli in order to encourage infants to continuously watch the stimuli to the end of the recording. That is, infants’ habituation to the stimuli that might lead to looking away from the screen was avoided.

The visual stimuli consisted of three novel cartoon animals (called A1, A2, and A3; see Fig. 1) presented in animated clips created with the Animation:Master software (Hash, Inc). Each of the animals occupied an area of approximately 12° × 9° (width × height) on the screen. The auditory stimuli (16 bit, mono, 22050 Hz digitization) were based on three animal sounds: a peacock call (a long continuous howl, S1), prairie dog barks (five short barks, S2), and a sea lion call (three short calls, S3), downloaded from www.findsounds.com. The sounds were processed by changing their frequencies so as to maintain their acoustic complexity while making them unlike any existing animal sound. The amplitudes of the sounds were equalized with the same total root mean square (RMS) at the level of −16.2 dB. The duration of the sounds was edited to be 2000 ms. The sounds were presented over a pair of loudspeakers located at either side of the monitor behind a black curtain. The loudness of the sounds was 52 dB SPL. The background noise in the chamber was 38 dB SPL.

### Design and Procedure

Prior to familiarization a 9-point calibration sequence was used to calibrate the remote eye tracker (sampling frequency 120 Hz, system accuracy 0.5 degrees). During calibration, a small animated object with sound was displayed at 9 locations on the screen (left, center and right × top, middle and bottom row). Calibration was repeated up to 3 times, or until all 9 points had been calibrated successfully.

**Figure 4:**
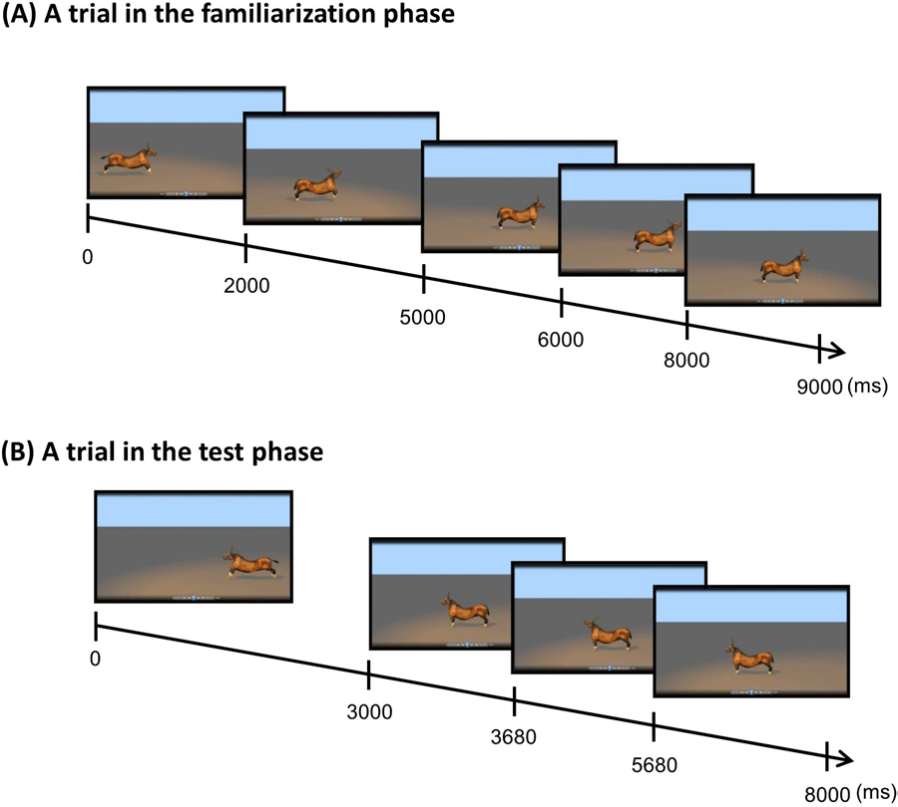
Animations used in the study. (**A**) Each familiarization trial consisted of a 9000 ms animation: either A1 ran, or A2 jumped, to the center of the screen from the left or right side for 5000 ms, raising its head to produce a call while moving. The animal then stopped in the center of the screen for 4000 ms and raised its head again to repeat the call. The call of each animal was therefore played twice in each familiarization trial, at 2000–4000 and 6000–8000 ms. (**B**) Each test trial consisted of an 8000 ms animation: The animal ran to the center of the screen from the right side for 3000 ms and stopped in the center of the screen for 5000 ms. Each animal raised its head and produced the call a single time at 3680–5680 ms. The animations were created using the Animation:Master software (Hash, Inc). Note that the time line in the figure is not scaled.

In the familiarization phase, half of the participants in each age group were presented with 9-sec clips with the animal-sound pairings of A1-S1 and A2-S2, and the other half with A1-S2 and A2-S1. In each clip, the animal started moving forward from either the left or right side of the screen toward the center (see Fig. 4A). Hence, there were four types of trial (two animals × two sides). These four trials comprised a block, and their presentation order in each block was pseudo-randomized. An inter-trial interval with a black screen was presented for 500 ms. There were four blocks in the familiarization phase; that is, 16 trials with 8 for each audiovisual pair. Each block was separated by a 3-sec attention getter movie. Each attention getter consisted of a colorful image of a cartoon animal which was moving or jittering slightly and was paired with a brief electronic sound.

In the test phase, each clip lasted for 8 sec. Three types of trials were presented (Fig. 4B). In the *familiar* trials, the animal-sound pairs were the same as trained in the familiarization phase. In the *switched* trials, the sounds paired with each animal were swapped. The presentation order of these four trials (familiar/switched × A1/A2) was counterbalanced between participants. Finally, a novel trial with a new animal (A3) producing a new sound (S3) was presented. The whole session took about 5 minutes to complete.

### Data analysis

The total looking time in each familiarization and test trial was derived from the sum of the duration that the infant was watching the video (i.e., the area of interest covered the whole screen) using Tobii studio software (version 3.4.5). The proportion of looking time was calculated in terms of the total looking time in a given trial divided by the duration of that trial.

Participants’ pupil diameter of both eyes was recorded when watching the video of each test trial (maximum 960 samples) and analyzed using Matlab with the following procedure:

1. Inclusion criterion: in a trial, if the missing rate of pupil diameter data was lower than 40%, the trial was included for further analysis (72/90 trials were included for the 10-month-olds, and 60/80 trials were included for the 15-month-olds). For each participant, there had to be at least one trial remaining in each familiar, switched, and novel condition. As a result, there were 14 10-month-olds and 11 15-month-olds in the final analysis.
2. Interpolating missing data: when there was a gap in the pupil diameter data where the eye tracker had not recorded the eyes, a linear interpolation was used to connect the last sample before the break and the first sample after the break. Most of the interpolated gaps were shorter than 500 ms (99.1% in 10-month-olds, and 98.9% in 15-month-olds). Hence, the length of the interpolated gaps was fitted with an exponential function in each age group. The mean length of the interpolated gaps was 32.3 ms (95% CI = [31.4, 33.3]) in 10-month-olds, and 37.4 ms (95% CI = [36.3, 38.6]) in 15-month-olds.
3. 4-Hz low-pass filter: Given the fact that pupillary responses would not change direction (either expand or constrict) with a frequency of higher than 4 Hz (every 250 ms)^36^, a 4-Hz low-pass filter was used to smooth the pupil diameter data in order to reduce recording noise.
4. Pupil data from both eyes in each sample were averaged.
5. Baseline correction: in order to correct for individual differences in pupil size, the mean pupil diameter in the 1000 ms time-window before the sound onset in each test trial was subtracted from the pupil diameter in each sample of the test trial for each individual participant.
6. 50 ms time bin: there were 520 samples (4,320 ms) of corrected pupil diameter data after the sound onset. Every 6 samples were then averaged to represent the pupil diameter in a 50-ms time bin. This resulted in 86 time bins, and the last 4 samples at the end of the movie were discarded (so that last time bin ended at 4,300 ms after sound onset).
7. The data of each time bin were submitted to a repeated-measures one-way analysis of variance (ANOVA) with three levels: familiar, switched, and novel. The effect of a time bin was considered significant only when its one preceding and following time bins were both significant as well (i.e., at least three successive time bins covering a range of 150 ms were significant). The effect size, ${\eta }_{p}^{2}$, was used to describe the proportion of the total variability attributed the factor of these three conditions^37^. Subsequently, at the time bins where the *F*-test was significant, *post*-*hoc t*-tests with Bonferroni correction (two-tailed, *p* < 0.05) were used. The effect size Hedges’ *g*_*av*_ was used. This is the effect size that is based on the same idea as Cohen’s *d* (i.e., the standardized mean difference of an effect), but the bias attributed to small sample size, and the correlation between measures in the within-participant design, are corrected^29^. The effect size Hedges’ *g*_*s*_, was used when the compared conditions was in the between-participant design instead.
8. Due to the multiple comparisons across 86 time bins, the family-wise Type-I error rate of these *F*-tests and t-tests may be inflated above the critical level (*α* = 0.05). The solution of this multiple comparison problem was inspired by a nonparametric statistical test that is now commonly used in the time-series data in EEG and MEG studies^38^. The null hypothesis of this test is that the data in different experimental conditions are drawn from the same probability distribution; in other words, the generated *p* value represents the likelihood that the sampled data in different condition originate from the same population. See Supplementary Materials for the procedure of this nonparametric statistical test. The reported time window for each effect in the Results was based on the outcomes of the nonparametric statistical tests.

## Electronic supplementary material

SUPPLEMENTARY MATERIALS
