# Supplementary material for: Different novelties revealed by infants’ pupillary responses
Source: Sci Rep. 2018 Jun 22;8:9533. doi: 10.1038/s41598-018-27736-z (PMC6015008; doi:10.1038/s41598-018-27736-z)
Supplement: Supplementary file 1 — SUPPLEMENTARY MATERIALS [file 41598_2018_27736_MOESM1_ESM.docx]

**SUPPLEMENTARY MATERIALS**

**Different novelties revealed by infants’ pupillary responses**

Yi-Chuan Chen and Gert Westermann

**Multiple comparisons using nonparametric statistical tests**

The non-parametric method was conducted in order to determine the time-window of each effect (see Figure S1). The time-window of the main effect between familiar, switched, and novel conditions was examined first. For each participant, the observed value in each condition among the time bins at which the main effect was significant (the light-grey area in Figure S1, see Step 7 in the Data Analysis section) was randomly selected to represent the individual’s performance in that condition. Then, these randomly sampled data were submitted to a one-way analysis of variance (ANOVA) and the main effect was examined using *F*-test. After repeating such random sampling and *F*-test for 1000 times, the Monte Carlo *p*-value was estimated based on the percentage that the *F*-tests failed to reach significance (*p* < .05) among these 1000 times. The Monte Carlo *p*-value smaller than 0.05 suggests that the possibility concerning to the data in the familiar, switched, and novel conditions originating from the same population is smaller than 5%. The Monte Carlo *p*-values were estimated for 10 times repeatedly in order to ensure that the Type-I error (α) was reliably controlled under the 0.05 level (i.e., Monte Carlo *p*-value < .05). If failed, the first and last time bins was eliminated (the dark-grey area in Figure S1), and the above procedure was repeated until the criterion was satisfied. At this step, the time-window of the main effect was determined.

Then, each paired comparison between familiar, switched, and novel conditions was conducted using the same procedure but the *F*-test was replaced with a paired *t*-test with Bonferroni correction. For example, the time-window of the perceptual novelty effect between familiar and novel conditions was examined by randomly sampling the data in these two conditions among the significant time bins and submitted to a *t*-test. Given that this is a multiple comparison and the α-level needs to be well controlled, the Monte Carlo *p*-value was estimated based on the percentage that the *t*-tests failed to reach significance (*p* < .017) among the 1000 times of sampling. The time-window was determined when the Monte Carlo *p*-value was smaller than 0.05 repeatedly for 10 times.

**Figure S1. The procedure of the non-parametric statistical tests.**
